# Synchronous occurrence of two lateral periodontal cysts in the same patient. Report of a rare case and review of the literature

**DOI:** 10.4317/jced.56802

**Published:** 2020-04-01

**Authors:** Ilias Karveleas, Eleni-Marina Kalogirou, Konstantinos I. Tosios, Nikolaos G. Nikitakis

**Affiliations:** 1 DDS, Private Practice, Athens, Greece; 2 DDS, MSc, PhD Candidate, Department of Oral Medicine and Pathology, Faculty of Dentistry, National and Kapodistrian University of Athens, Greece; 3 DDS, PhD, Associate Professor, Department of Oral Medicine and Pathology, Faculty of Dentistry, National and Kapodistrian University of Athens, Greece; 4 MD, DDS, PhD, Professor and Chair, Department of Oral Medicine and Pathology, Dental School, National and Kapodistrian University of Athens, Greece

## Abstract

We present a case of a patient with two lateral periodontal cysts in the maxilla and the mandible, respectively, and review the English literature on multiple lateral periodontal (LPCs) cysts and/or gingival cysts (GCs) and botryoid odontogenic cysts (BOCs). The patient was a 59 year-old female with two fluctuant swellings covered by semi-lucent mucosa on the attached gingiva between the maxillary and mandibular right canine and first premolar teeth, respectively. Periapical radiographs revealed at the respective sites between the roots of the canine and first premolar teeth areas unilocular radiolucencies. Intra-operatively, the presence of bone cavities was confirmed at both sites. The microscopic features were consistent with LPC. The review of the English literature on multiple LPCs and/or GCs and BOCs found seven reports of multiple LPCs, four of multiple GCs, and two with an LPCs and a GC. It is concluded that multiple LPCs have been rarely reported in the literature, but should be included in the differential diagnosis of multifocal radiolucencies lateral to vital teeth. The possibility of multiple lesions in different locations should direct to a thorough clinical and radiographic examination in a patient diagnosed with an LPC or GC.

** Key words:**Jaw cysts odontogenic cyst, lateral periodontal cyst, multifocal unilocular radiolucencies.

## Introduction

*Lateral periodontal cyst* (LPC) is a developmental odontogenic lesion that usually occurs in the anterior-premolar area of the mandible, lateral to the roots of vital teeth (1). Although its incidence is estimated to less than 1% of odontogenic cysts (2), it may actually be underdiagnosed rather than rare, as it is usually an incidental radiographic finding (3). Both genders are equally affected and most patients are in the fifth to seventh decades of life (1). It is usually described as a well-defined, round, oval or teardrop-shaped, corticated radiolucency laterally to or between the roots of vital teeth (2) that measures approximately 1cm in diameter (1). It commonly causes bone perforation (1) and clinically manifests as an asymptomatic gingival swelling of normal or bluish to gray color (4,5). Alveolar bone expansion is rare (2). The multicystic variant of LPC is termed *botryoid odontogenic cysts* (BOC) and its extraosseous counterpart *gingival cyst* (GC), previously referred to as gingival cyst of the adult (2).

The synchronous presence of LPCs in different sites of the same patient has been rarely documented (4-6). We present a case of a patient with two LPCs, one in the maxilla and one in the mandible, respectively, and review the English literature on multiple LPCs and/or GCs/BOCs.

## Case Report

A 59 year-old female was referred for diagnosis and management of two gingival swellings discovered during routine dental examination. The patient recalled that she had first noticed them more than 6 years ago, but as they did not cause any aesthetic or functional disturbance she neglected to ask for medical advice. Her medical history was non-contributory and a recent complete blood count was within normal limits.

**Figure 1:**
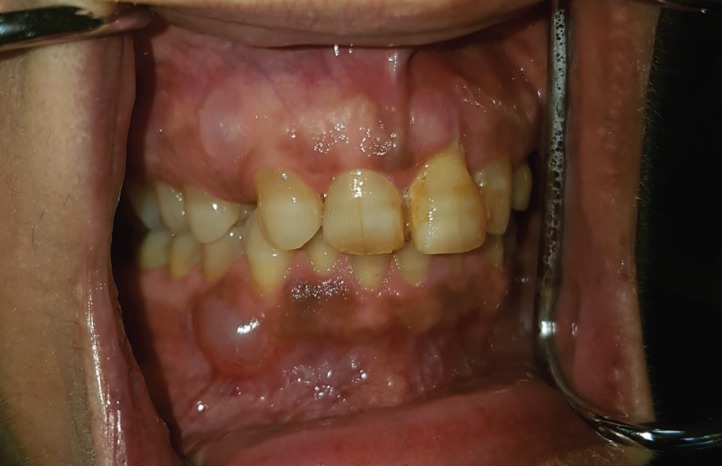
Two discrete swellings covered by normal to slightly semi-slucent mucosa on the attached gingiva between the maxillary and mandibular right canine and first premolar teeth.

**Figure 2:**
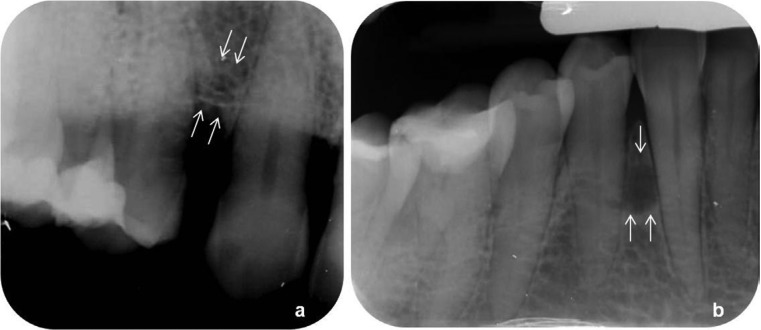
Periapical radiographs revealed radiolucencies between (a) the maxillary and (b) the mandibular right canine and first premolar teeth.

Intraoral examination revealed two fluctuant swellings covered by semi-lucent mucosa on the attached gingiva between the maxillary and mandibular right canine and first premolar teeth, respectively, each measuring approximately 1cm in diameter (Fig. 1). The adjacent teeth were vital with a normal gingival sulcus depth and no bleeding on probing. Both maxillary lateral incisors were congenitally missing, while the right first premolar had a lateral-access class II amalgam restoration. Periapical radiographs revealed at the respective sites between the roots of the canine and first premolar teeth areas unilocular radiolucencies (Fig. 2). No other abnormality was clinically or radiographically evident.

**Figure 3:**
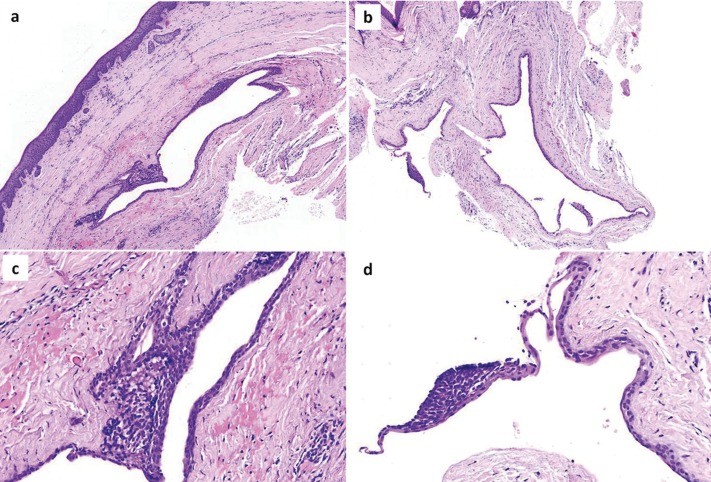
Microscopic examination of (a) the maxillary and (b) mandibular lesion showed cystic cavities lined by non-keratinized, cuboidal to stratified squamous epithelium. Higher magnification shows focal nodular thickenings rich in clear cells in the lining epithelium of (c) the maxillary and (d) the mandibular cyst (hematoxylin and eosin stain, original magnifications A, B x25; C, D x200).

With the working diagnosis of developmental odontogenic cysts, in particular LPCs or odontogenic keratocysts (OKCs), surgical excision was performed under local anesthesia. Intra-operatively, the presence of bone cavities was confirmed at both sites. Grossly, the lesions had a tan to brown cut surface. Microscopic examination of 5μm thick formalin-fixed and paraffin-embedded tissue sections showed that the cystic wall was lined by non-keratinized, cuboidal to stratified squamous epithelium (Fig. 3), presenting focal nodular thickenings rich in clear cells that were Periodic Acid-Schiff positive. The connective tissue wall showed subepithelial hyalinization and islands of odontogenic epithelium. The clinical, radiographic, intraoperative and microscopic features in both cases were consistent with LPCs.

The postoperative course was uneventful and no recurrence has been recorded during the 18-month follow-up period. At the time of initial examination, the patient gave informed consent for the future use of her data for study.

## Discussion

The cysts presented herein could be LPCs perforating the cortical bone of the jaws and extending beneath the oral mucosa, or GCs causing bone erosion. The differentiation between LPC and GC is not always straightforward, as both lesions share many clinical features such as anatomic site, may appear as a gingival swelling, and have identical pathology (1). Direct contact of the cyst with the periodontal ligament of a tooth, seen radiographically as a well-defined radiolucency along the root surface or detected intra-operatively (7), as well as presence of a bone cavity with smooth borders (8), have been considered suggestive of LPC. In our case, the diagnosis of LPCs was favored, as two well-defined bone cavities with smooth borders were found intraoperatively, not consistent with superficial erosion or saucerization of the cortical plate (1,2). It should be noticed, however, that most authors suggest that LPC and GC represent the central and peripheral counterpart of the same entity and have no differences in treatment and prognosis (1,7,9,10), therefore their differentiation may be considered as an academic exercise.

Our case is unusual in that two LPCs were found in the same patient, one in each jaw. A review of the English literature on multiple LPCs and/or GCs and BOCs was performed through MEDLINE/PubMed and Google Scholar databases until November 17, 2019 with the keywords: *multiple lateral periodontal cysts, multifocal lateral periodontal cysts, multifocal botryoid odontogenic cysts, multiple botryoid odontogenic cysts, multiple gingival cysts, multifocal gingival cysts*. Inclusion criteria were occurrence of more than one LPC, GC, and BOC in the same patient, with radiographic and histopathological documentation (2).

There were seven reports of multiple LPCs,(4-6) four of multiple GCs (7,9,11,12), two with an LPC and a GC (3,13), but none of multiple BOCs or a BOC along with LPC or GC in the same patient (Table 1). The case of “multiple LPCs (botryoid variant), or botryoid odontogenic cyst” was included in our review, as microscopically the two cysts were found to be separated by 4mm of bone (6). The case of Legunn (14) in a 67-year old male with two lesions clinically and radiographically consistent with LPCs was excluded, as there was no microscopical confirmation of the diagnosis in one of them. Case 2 of Carter *et al.* (15) with one LPC in the anterior and one in the molar region of the mandible that was included in their Table, but was not further mentioned in the text or included in the diagram of the site distribution was, also, excluded. The case of Govil *et al.* (16) was not included as the clinical and microscopical features are more consistent to inflammatory cysts. Finally, the multiple gingival cysts reported by Fardal and Johannessen (17) are consistent with peripheral OKCs in a patient with Nevoid Basal Cell Carcinoma Syndrome (NBCCS).

**Table 1:**
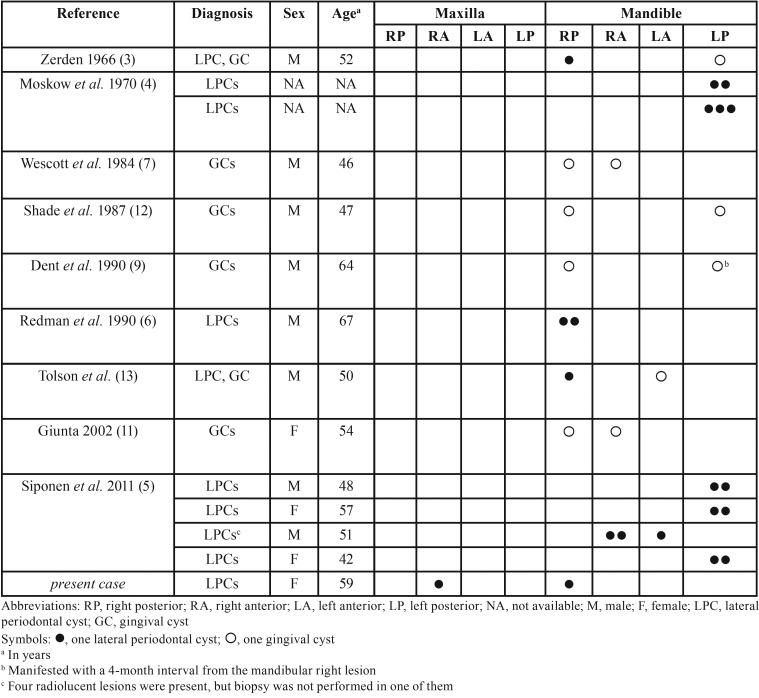
Demographics and location of previous cases of multiple lateral periodontal cysts/ gingival cysts and the present case.

The main clinical features of previously reported cases of multiple LPCs, GCs, or their combination, as well as the present one are summarized in Table 1. Multiple LPCs involved three males and three females, with a mean age of 54±8.85 years (Table 1). Information about the race was available in two cases; one patient was Caucasian (present case) and one black (6). Three cases, including the present one, manifested as gingival swellings (5,6). Only one patient complained for “sensitivity” in the area of LPCs (5). In six cases, including ours, there were two LPCs per patient (4-6), whereas two patients presented with three LPCs each (4,5). Six patients exhibited multiple LPCs unilaterally in the mandible (4-6), while a 51-year old male had LPCs in both right and left anterior mandibular areas (5). Our case is unique in that one lesion was located in the maxilla that was not involved in previous cases with multiple LPCs and is the site of less than 1/3 of solitary LPCs (1). As in our LPCs, in most previous cases the radiolucencies were associated with the middle or apical segment of the root (4-6), while relation to the cervical segment, close to the bone edge, was found in only one case (4).

Two GCs in the same patient have been described in three males and one female of African descent, with a mean age of 52.75±8.3 years (7,9,11,12). In one case, the second GC presented metachronoulsy, with a four-month interval (9). Multiple GCs appeared as painless, normal-colored to yellow-pink gingival nodules measuring 0.3 to 0.6 cm in diameter and were not associated with radiographic findings (7,9,11,12). All cases involved the mandibular area from lateral incisor to first premolar, unilaterally (7,11) or bilaterally (9,12).

Synchronous occurrence of a LPC and a GC have been described by Tolson *et al.* (13) in a 50 year-old black male, located between the mandibular right premolars and lateral to the left lower lateral incisor, respectively, and by Zerden (3) in a 52 year-old black male, between the lower right premolars and the left mandibular canine and premolar, respectively.

In the case reported herein, the clinical and radiographic similarities between the maxillary and mandibular lesion focused the differential diagnosis on intraosseous entities that might appear as multifocal, unilocular radiolucencies, and manifest as gingival swellings. OKC is the second most common developmental odontogenic cyst, comprising 10-20% of odontogenic cysts; it has a predilection for the posterior mandible of young patients in the age range of 10-30 years or 50-70 years (2). OKC might be asymptomatic and randomly discovered on radiographic examination as periapical, periradicular or interradicular, unilocular or multilocular radiolucency,(18) or may cause jawbone expansion (2) or/and manifest as gingival swelling (18). Multiple OKCs in the same patient occur predominantly among individuals with NBCCS, an autosomal dominant inherited disease manifesting with cutaneous basal cell carcinomas, jaw OKCs and skeletal anomalies (19). OKC is often the first sign of the NBCCS in patients younger than 20 years old (19), but there are cases where an OKC led to the diagnosis of the syndrome in aged patients (20). Multiple OKCs may also present in non-syndromic patients (21). Adenomatoid odontogenic tumor (AOT) is a benign epithelial odontogenic tumor that arises intraosseously in association (follicular variant) or not (extrafollicular variant) with an unerupted tooth and can have a predominantly cystic component. Extrafollicular AOT shows predilection for females in the third decade of life, and, in contrast to the follicular subtype, involves both jaws in almost equal incidence. AOT might be asymptomatic or cause cortical bone perforation (22) and, thus an intraoral swelling (23). The most frequent radiographic appearance of AOT is a well-defined, unilocular radiolucency (22), often interradicular (24), while central lesional radiopacities might be also observed (24). A few cases of multiple AOTs in the same patient have been described in the literature (23), almost all of them of the follicular variant. As the lesions in the present case showed fluctuation on palpation, suggestive of cystic lesions, the possibility of an odontogenic tumor that may present multifocally, such as squamous odontogenic tumor (25), calcifying epithelial odontogenic tumor (26), central odontogenic fibroma (27), central ossifying fibroma (2), as well as central giant cell lesions (28) was discarded.

The origin of LPC and GC has been traced to odontogenic epithelial remnants, predominantly from the dental lamina and, less probably, from the reduced enamel epithelium or the rests of Malassez (2,10). As seen in Table 1, in 5 of 8 cases of multiple LPCs all cysts were clustered in the same area (4-6), indicating a common locally acting factor (10). Chance may account for cases like ours, where the cysts manifested in different anatomical locations, as the number of cases reported so far is too small. It is noteworthy that all previously reported cases with synchronous occurrence of LPCs/GCs with available information on race, manifested in black persons (3,6,7,9,11-13).

As in solitary cases, enucleation without removal of the adjacent teeth is the treatment of choice for multiple LPCs/GCs (4-7,9,11,12). Few studies have documented uneventful healing and the absence of recurrence of multiple LPCs/GCs (9,12), but follow up information was not available in most cases reviewed (4-7,11).

Multiple LPCs have been rarely reported in the literature, but should be included in the differential diagnosis of multifocal radiolucencies lateral to vital teeth. The possibility of multiple lesions in different locations should direct to a thorough clinical and radiographic examination in a patient diagnosed with a LPC or GC.
